# Three Dimensions of Precision in LetibotulinumtoxinA Treatment: Dose, Dilution and Injection Pattern in Relation to Diffusion

**DOI:** 10.3390/toxins18100408

**Published:** 2026-09-23

**Authors:** Maxine Bennek, Martina Kerscher

**Affiliations:** Department of Cosmetic Sciences, Institute of Biochemistry and Molecular Biology, Faculty of Mathematics, Informatics and Natural Sciences, University of Hamburg, 20146 Hamburg, Germany; martina.kerscher@uni-hamburg.de

**Keywords:** LetibotulinumtoxinA, spread, aesthetics

## Abstract

Botulinum neurotoxin type A (BoNTA) is widely used for the treatment of dynamic facial wrinkles, where precise control of toxin diffusion is essential to achieve predictable aesthetic outcomes while preserving natural facial expression. However, the relative influence of injection pattern, dose, and dilution on diffusion characteristics remains incompletely understood. In this exploratory case series, treatment-naïve subjects undergoing aesthetic treatment of horizontal forehead lines with LetibotulinumtoxinA (Leti-BoNTA) were assigned to one of four injection paradigms according to their wrinkle pattern: a conventional five-point injection pattern, the previously published LEBO injection pattern, dose variation (2–5 U per injection point), or dilution variation (reconstitution volumes 0.675–2.5 mL). Diffusion was assessed using the Minor iodine–starch test, and anhidrotic areas were quantified in cm^2^. Increasing dose was associated with progressively larger anhidrotic areas in this exploratory case series. Greater reconstitution volumes were associated with broader toxin distribution, whereas lower dilution volumes produced more confined diffusion areas. The conventional five-point injection pattern resulted in relatively extensive diffusion, whereas the LEBO protocol demonstrated a more localized and consistent distribution profile. Despite differences in diffusion characteristics, natural facial dynamics were preserved across all treatment paradigms. These findings suggest that injection pattern, dose, and dilution collectively influence Leti-BoNTA diffusion and should be considered when optimizing individualized treatment strategies.

## 1. Introduction

Botulinum neurotoxin type A (BoNTA) is one of the most widely performed non-surgical aesthetic treatments worldwide [1]. Its ability to temporarily reduce dynamic facial wrinkles by inhibiting acetylcholine release at the neuromuscular junction has made it a cornerstone of modern aesthetic medicine [2]. Despite its widespread clinical use, achieving predictable and natural outcomes requires a precise understanding of how injection techniques influence toxin distribution and muscular modulation [3].

In aesthetic treatments of the upper face, particularly the frontalis muscle, injection precision is essential to correct unwanted facial expressions while preserving natural facial dynamics [4]. Excessive toxin spread may lead to undesired effects such as brow ptosis or loss of expressive movement, whereas insufficient diffusion may result in suboptimal treatment outcomes [5]. Therefore, understanding the factors that influence toxin diffusion is critical for optimizing treatment protocols.

Several parameters may influence the spread and diffusion behavior of botulinum neurotoxin type A (BoNTA), including the injection pattern, the dose administered per injection point, and the dilution of the toxin preparation. Previous studies have suggested that these factors may affect the spatial distribution of the toxin within the treated muscle [3]. In addition, comparative investigations have shown that different BoNTA formulations may exhibit distinct diffusion characteristics. In a previous clinical investigation comparing OnabotulinumtoxinA, AbobotulinumtoxinA, and LetibotulinumtoxinA, Leti-BoNTA demonstrated a relatively confined and predictable diffusion profile while maintaining clinical efficacy, suggesting a high level of injection precision [6].

Despite these observations, the relative contributions of injection pattern, dose, and dilution to toxin diffusion and spread in clinical practice remain incompletely understood. In particular, the influence of modern anatomically guided injection protocols has received limited investigation. One such concept is the LEBO technique (Lateralization, Elevation, Balancing and Optimization), an anatomically guided injection approach that aligns injection points with the line of convergence of the frontalis muscle. This strategy aims to optimize eyebrow elevation, balance muscular activity, and minimize undesired toxin spread while preserving natural facial dynamics [7]. Further investigation of these parameters may therefore help refine injection strategies aimed at achieving precise wrinkle reduction while maintaining physiologic facial expression.

The exploratory objective of this study was to investigate how different injection parameters influence the diffusion and spread, as well as functional effects, of Leti-BoNTA. Four injection paradigms were evaluated addressing three key treatment variables: injection pattern, dose, and dilution.

## 2. Results

A total of twelve participants aged 23–59 years were included in this exploratory case series, with three subjects assigned to each of the four injection paradigms. All twelve participants received the assigned intervention and completed the scheduled follow-up visits and diffusion assessments. No participants were lost to follow-up, and all twelve participants were included in the descriptive analysis.

Given the exploratory design of this investigation with three subjects per protocol, data are presented descriptively as means and standard deviations. The reported differences between protocols and treatment groups reflect observed trends rather than statistically confirmed effects.

In Case 1, increasing injected units per site (from 2 U to 5 U) was associated with progressively larger anhidrotic areas. The smallest dose (2 U) produced the most restricted spread, while the highest dose (5 U) consistently generated the largest areas of anhidrosis (Figure 1), consistent with a possible dose-related trend in this small case series. Mean anhidrotic areas increased from 14.49 ± 3.24 cm^2^ (2 U) to 32.61 ± 2.88 cm^2^ (5 U) at day 28, with a comparable pattern at day 57 (10.88 ± 3.87 to 27.70 ± 3.68 cm^2^) and day 112 (6.58 ± 3.02 to 19.76 ± 1.44 cm^2^) (Table 1).

In Case 2, a general trend towards larger anhidrotic areas was observed with increasing reconstitution volume/injection volume per point. Smaller reconstitution volumes (0.675 mL/50 U) produced the most confined distribution, whereas larger volumes (1.25–2.5 mL/50 U) typically resulted in a wider spread. Despite inter-individual differences in absolute anhidrotic area size, increasing reconstitution volumes consistently resulted in a broader functional spread in all subjects (Figure 2). Mean anhidrotic areas ranged from 25.35 ± 0.53 cm^2^ (0.675 mL) to 50.19 ± 6.81 cm^2^ (2.5 mL) at day 28, from 19.53 ± 1.64 to 38.33 ± 3.17 cm^2^ at day 57, and from 8.24 ± 3.39 to 26.00 ± 1.61 cm^2^ at day 112 (Table 2).

In Case 3, treatment with 20 U Leti-BoNTA resulted in relatively large anhidrotic areas at early follow-up. The mean diffusion area was 134.94 ± 33.55 cm^2^ at day 28, decreasing to 126.29 ± 31.79 cm^2^ at day 57, and to 93.14 ± 14.84 cm^2^ at day 112, with a comparatively wide inter-subject range at each timepoint. Inter-individual variability was observed, particularly during the early follow-up visits (Figure 3).

In Case 4, treatment with 16 U Leti-BoNTA produced more confined anhidrotic areas. The mean diffusion area was 111.17 ± 6.96 cm^2^ at day 28, 98.90 ± 7.67 cm^2^ at day 57, and 88.48 ± 9.43 cm^2^ at day 112, with a notably narrower standard deviation than that observed for Case 3 at each corresponding timepoint (Table 3).

Compared with the five-point injection pattern, the LEBO [7] approach showed a more localized diffusion. Furthermore, visual inspection of the diffusion profiles suggested lower inter-individual variability with the LEBO scheme, resulting in a more homogeneous treatment response across subjects. In contrast, the five-point injection pattern appeared to be associated with greater variability in the extent of anhidrosis (Figure 4).

These findings support a dose–response relationship under otherwise identical injection conditions. Despite the spread-related differences in anhidrotic areas, all participants maintained recognizable facial dynamics during maximum frown, suggesting that Leti-BoNTA allows functional preservation of facial dynamics while reducing visible wrinkles. As this assessment was based on visual evaluation, it should be regarded as a supportive qualitative observation. No adverse events were reported by any participant throughout the follow-up period.

## 3. Discussion

The present exploratory case series investigated how different injection parameters—specifically injection pattern, dose, and dilution—affect the diffusion characteristics and spread of Leti-BoNTA and their potential impact on facial mimics and facial dynamics. The results demonstrate that diffusion behavior is influenced by several controllable treatment variables, highlighting the importance of precise injection planning in aesthetic neuromodulator therapy.

When comparing the two injection patterns, the LEBO protocol resulted in a more confined diffusion profile than the conventional five-point injection pattern. While the five-point pattern showed larger anhidrotic areas, the LEBO [7] approach demonstrated a more localized and consistent spread across all subjects. Furthermore, the findings suggest that anatomically guided approaches may support not only a more confined diffusion profile but also greater consistency of toxin distribution between subjects. Accordingly, anatomically guided injection concepts, such as the LEBO scheme, may represent a valuable alternative to conventional injection protocols in daily clinical practice. By aligning injection points with the natural line of convergence of the frontalis muscle, the LEBO [7] technique may allow improved targeting of muscle activity while limiting unintended toxin spread. The biomechanical model underlying the line of convergence [8] has more recently been complemented by computational work proposing a different contraction pattern for the frontalis muscle [9]. As this is an evolving area of research, the anatomical basis of the LEBO technique should be viewed within this broader context.

Precision is a key factor in aesthetic neuromodulator treatments [10]. The ability to correct unwanted facial dynamics while preserving natural facial dynamics represents one of the main goals of modern aesthetic medicine [11]. Previous work by Bennek et al. (2025) [6] demonstrated that Leti-BoNTA provides effective neuromodulation while maintaining natural-looking facial dynamics. In agreement with these findings, recognizable facial expressions during maximum frown were observed across all injection paradigms evaluated in the present study (Figure 5), despite measurable differences in diffusion patterns. This suggests that the observed variations in toxin spread were not associated with a loss of subtle facial movement or facial expressivity.

Beyond clinical observation, recent computational modelling approaches have sought to characterize BoNTA diffusion and immunogenicity in silico. A multiscale simulation across eight BoNTA formulations over a 20-year horizon predicted that Leti-BoNTA combined high efficacy with the most confined diffusion profile and lowest modelled immunogenicity among the formulations evaluated [12]. A separate targeted in silico comparison of Leti-BoNTA and OnabotulinumtoxinA similarly reported a smaller effective diffusion radius and more rapid immune clearance for Leti-BoNTA under matched conditions [13]. Both modelling studies rely on mechanistic simulation rather than direct clinical measurement, and their authors explicitly characterize the findings as hypothesis-generating; nonetheless, they are broadly consistent with the confined diffusion pattern observed empirically in the present investigation.

Together with the findings from the injection-pattern experiments, the dose variation results suggest that the extent of anhidrotic spread may be protocol-dependent rather than a fixed characteristic of Leti-BoNTA. This observation is broadly consistent with previously reported dose–response relationships for botulinum toxin formulations, where higher doses have been associated with greater functional effects and wider areas of muscular modulation.

For example, Carruthers and Carruthers [14] demonstrated that increasing doses of botulinum toxin were associated with greater clinical efficacy and more pronounced muscle relaxation in aesthetic treatments of the upper face. These findings support the concept that careful dose titration is essential to balance treatment efficacy with safety.

Similarly, the dilution variation paradigm demonstrated that higher reconstitution volumes were associated with broader diffusion patterns, whereas smaller dilution volumes resulted in more confined areas of anhidrosis. These findings suggest that dilution represents an additional parameter that can be used to modulate toxin spread. From a clinical perspective, lower dilution/injection volumes may therefore enable higher precision when treating delicate facial areas where unintended diffusion should be minimized. Conversely, broader diffusion patterns achieved through higher dilution/injection volumes may be advantageous when wider muscular coverage is desired. Whether lower dilution/injection volumes translate into greater clinical precision, or whether broader diffusion meaningfully benefits wider muscular coverage, remains a hypothesis that this small case series cannot directly confirm and that would require dedicated comparative studies to establish. These observations are consistent with the findings of Hsu et al., who reported that injections of botulinum toxin at lower concentrations and higher volumes resulted in greater diffusion and larger affected areas. Their results indicate that dilution and injection volume may influence the spatial distribution of the toxin within the treated muscle [15].

In contrast, Carruthers et al. were unable to detect a significant difference in treatment outcomes across a fivefold dilution range when treating the lateral orbital area. However, the authors acknowledged the limited sample size of their study and noted that dilution may still influence treatment results [16]. These differing findings highlight that the impact of dilution on toxin spread may depend on anatomical treatment area, injection technique, and study design.

Taken together, these findings support a treatment-planning framework in which formulation characteristics and injection technique are considered jointly, allowing practitioners to tailor the spatial distribution of the pharmacodynamic effect.

Several limitations should be considered when interpreting the results. The present study represents a small exploratory case series with only three participants per injection paradigm (N = 3 per group, N = 12 total), which substantially limits statistical power and the generalizability of the observed trends; findings should therefore be regarded as hypothesis-generating rather than confirmatory. In addition, diffusion was assessed indirectly through anhidrotic areas using the Minor iodine–starch test, which serves as a surrogate marker for toxin spread. Furthermore, interpretation of the Minor iodine–starch test involves a degree of subjectivity, and inter- or intra-rater reliability was not formally assessed in this study.

Larger controlled studies are warranted to further validate the observed diffusion patterns and to investigate their clinical implications in broader patient populations. Furthermore, this sample size did not permit inferential statistical testing; reported values represent descriptive summary statistics (mean ± SD) rather than confirmed statistical differences. In addition, Protocols 3 and 4 differed not only in injection geometry but also in total administered dose (20 U vs. 16 U) and number of injection points, which may have contributed to the observed differences in diffusion area independent of injection pattern.

## 4. Conclusions

This exploratory case series demonstrates that the diffusion characteristics of Leti-BoNTA are influenced by controllable treatment parameters, including injection pattern, dose, and dilution. Across the evaluated paradigms, modifications of these variables were associated with measurable differences in the spatial extent and consistency of the pharmacodynamic effect, suggesting that toxin spread may be influenced by treatment protocol rather than representing a fixed product characteristic.

Both dose and dilution appeared to modulate diffusion behavior, with higher doses tending to increase the functional diffusion area and larger reconstitution volumes resulting in broader distribution patterns even at constant unit amounts. In contrast, the anatomically guided LEBO [7] injection pattern produced a more localized and consistent diffusion profile than the conventional five-point approach, underscoring the importance of injection geometry in treatment planning.

Importantly, facial dynamics remained preserved in all subjects treated with 14–20 U of Leti-BoNTA, suggesting that effective neuromodulation can be achieved without compromising natural facial expression. These findings support a treatment-planning approach in which formulation characteristics and injection technique are considered jointly. For forehead treatment, protocol selection may therefore be understood as balancing precision of effect against the desired extent of muscular coverage. Additionally, Protocols 3 and 4 differed not only in injection pattern but also in total administered dose (20 U vs. 16 U) and number of injection points, which represents a confounding factor that cannot be fully disentangled from the effect of injection pattern itself.

Taken together, the present findings support a practical three-dimensional framework for individualized Leti-BoNTA treatment planning based on dose, dilution, and injection pattern. By adjusting these parameters, clinicians may tailor the spatial distribution of the pharmacodynamic effect and optimize treatment outcomes while minimizing unintended toxin spread.

## 5. Materials and Methods

The study was conducted in accordance with the Declaration of Helsinki and Good Clinical Practice (GCP) guidelines. The research protocol was reviewed and approved by the Ethics Committee of the Medical Faculty of Ludwig-Maximilians-Universität München, Germany. The clinical trial was registered in the Clinical Trials Information System (CTIS) under EU CT number 2024-511047-26-01 on 23 March 2024. All participants provided written informed consent prior to participation in the study. Written informed consent for the publication of study data and anonymized photographic material was obtained from all participants.

Patients included in these case reports were healthy male and female subjects aged 18–75 with a BMI of 16–27 kg/m^2^, a healthy skin test area with uniform sweating activity, an intended wish to get their horizontal forehead lines treated, and no areas of anhidrosis under standardized sweating conditions, which were provoked on a mechanical treadmill [6], assessed during the screening period (day 14 to day 1). As for all toxin injections in aesthetic treatments, careful subject selection is essential. The main inclusion and exclusion criteria were defined in accordance with previously established criteria described by Bennek et al. [6]. LetibotulinumtoxinA (Letybo^®^; Croma-Pharma GmbH, Leobendorf, Austria) was used for injection.

Participants were required to have symmetric dynamic forehead lines and to be naive to prior BoNTA treatment. Four different injection paradigms were evaluated to investigate the influence of dose, dilution, and injection pattern on the diffusion characteristics of Leti-BoNTA. These included a dose variation protocol (Case 1), a dilution variation protocol (Case 2), a conventional five-point injection pattern (Case 3), and the LEBO injection pattern [7] (Case 4). Each injection paradigm consisted of three subjects (N = 3) with injections administered into the frontalis muscle. Participants were assigned to one of the four injection paradigms based on their individual forehead wrinkle pattern and the suitability of the respective injection paradigm. Given the exploratory nature of the study, no formal sample size calculation was performed. The sample size was selected to allow an initial descriptive evaluation of diffusion patterns across the four injection paradigms.

The study was conducted at the Department of Cosmetic Sciences, University of Hamburg, Hamburg, Germany.

Cases 1 and 2 were specifically designed to investigate the influence of dosing and dilution on diffusion characteristics of Leti-BoNTA, whereas Cases 3 and 4 evaluated the impact of different injection patterns.

In Case 1, intramuscular injections of Leti-BoNTA were administered at four standardized injection points in the frontalis muscle with varying doses of 2 U, 3 U, 4 U, and 5 U per injection site while maintaining a constant reconstitution volume. This allowed the evaluation of the relationship between the injected dose and the observed anhidrotic response as an indirect indicator of functional toxin spread.

In Case 2, a constant dose of 4 U per injection point was administered across four standardized frontalis injection sites using different reconstitution volumes, reconstituted with a 50 U vial (0.675 mL, 1.25 mL, 2.0 mL, and 2.5 mL). This allowed the evaluation of the effect of dilution and injection volume on toxin spread while maintaining a constant unit dose.

In Case 3, a conventional five-point injection scheme was used for treatment of the frontalis muscle. Injection points were distributed symmetrically across the upper forehead to provide a standardized treatment approach commonly used in aesthetic practice, injecting 0.1 mL for each point (50 U vial reconstituted with 1.25 mL sodium chloride solution).

In Case 4, injections were performed according to the LEBO-based concept [7], which incorporates the line of convergence of the frontalis muscle to guide injection placement. Injection points were positioned along this anatomical reference line to balance eyebrow elevation and optimize muscular activity while minimizing the risk of brow descent.

Along this line, six deep intramuscular injections of 2 U Leti-BoNTA were administered bilaterally at the level of the medial brow, brow apex, and lateral brow. In addition, one superficial balancing point (1 U) was injected approximately 1.5–2 cm above the eyebrow. Further intramuscular optimization points (1 U each) were placed in areas of highest muscular activity when required.

An overview of the four injection paradigms is provided in Table 4.

Injection technique and anatomical placement were standardized across all cases to minimize procedural variability. On day 1, intramuscular injections were applied perpendicular to the skin surface using a 30-G needle along the mid-pupillary line, approximately 3 cm above the orbital rim. In relation to the line of convergence (C-line) [8], a template was used to ensure consistent placement of the injection points between all Cases (Figure 6, Figure 7, Figure 8 and Figure 9).

Study outcomes included the extent of the anhidrotic area (cm^2^) following Leti-BoNTA injection, assessed using the Minor iodine–starch test as an indirect marker of functional toxin spread. In addition, preservation of facial dynamics was evaluated qualitatively using standardized facial photography at rest and during maximum frown.

Diffusion was assessed using the Minor iodine–starch test, allowing visualization of toxin-induced anhidrotic areas by blocking the cholinergic synaptic sweat glands as described by Bennek et al. [6,17]. After the application of iodine and starch, optimal development of the characteristic dark-blue coloration was awaited during exercise on a mechanical treadmill before documenting the anhidrotic halos using standardized digital photography (VisioFace^®^ 1000D, Courage + Khazaka electronic GmbH, Cologne, Germany). The anhidrotic areas were subsequently measured and quantified in square centimeters (cm^2^) using a standardized image analysis program as an indirect indicator of toxin spread. To minimize potential measurement variability, standardized imaging conditions were maintained; however, factors such as camera angle, parallax effects, lighting conditions, and the absence of calibration markers or pixel-density control were considered possible sources of minor variability. Following documentation, residual starch was removed using an alcohol swab.

The Minor iodine–starch test was performed at baseline (day 1, prior to injection) and during follow-up visits on days 28, 57, and 112. In addition, functional outcomes were evaluated using standardized facial photography obtained at rest and during maximum frown to assess potential effects on facial dynamics. Image analyses and photographic assessments were performed by a trained evaluator experienced in aesthetic assessment who was not involved in the injection procedures. The evaluator was blinded to the injection paradigm during image analysis to minimize assessment bias.

Given the exploratory nature of the study and the small sample size of three subjects per injection paradigm, no inferential statistical analyses were performed. Data were analyzed descriptively and are presented as mean ± standard deviation (SD). No imputation of missing data was required, as all participants completed the scheduled follow-up assessments.

## Figures and Tables

**Figure 1:**
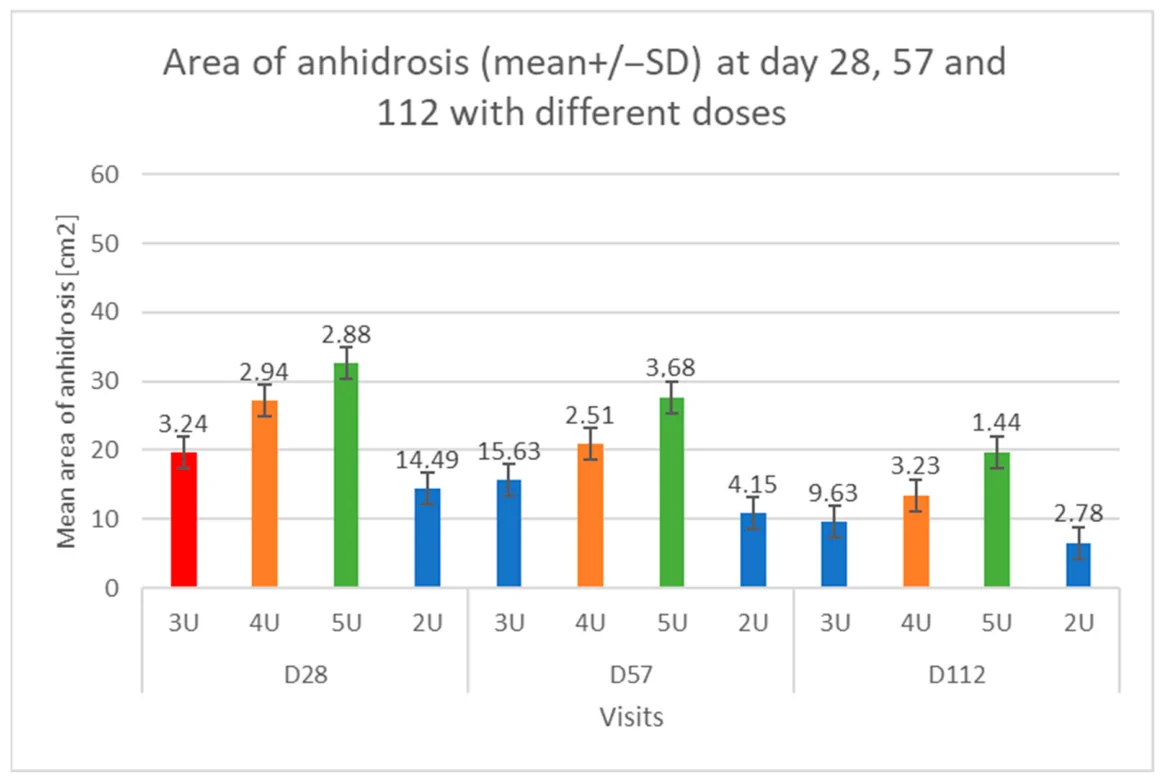
Dose Variation: Area of anhidrosis (mean ± SD, *n* = 3 subjects per dose) at day 28, 57 and 112 with injected doses of 2, 3, 4, and 5 U Leti-BoNTA per injection point (4 injection points, constant reconstitution volume).

**Figure 2:**
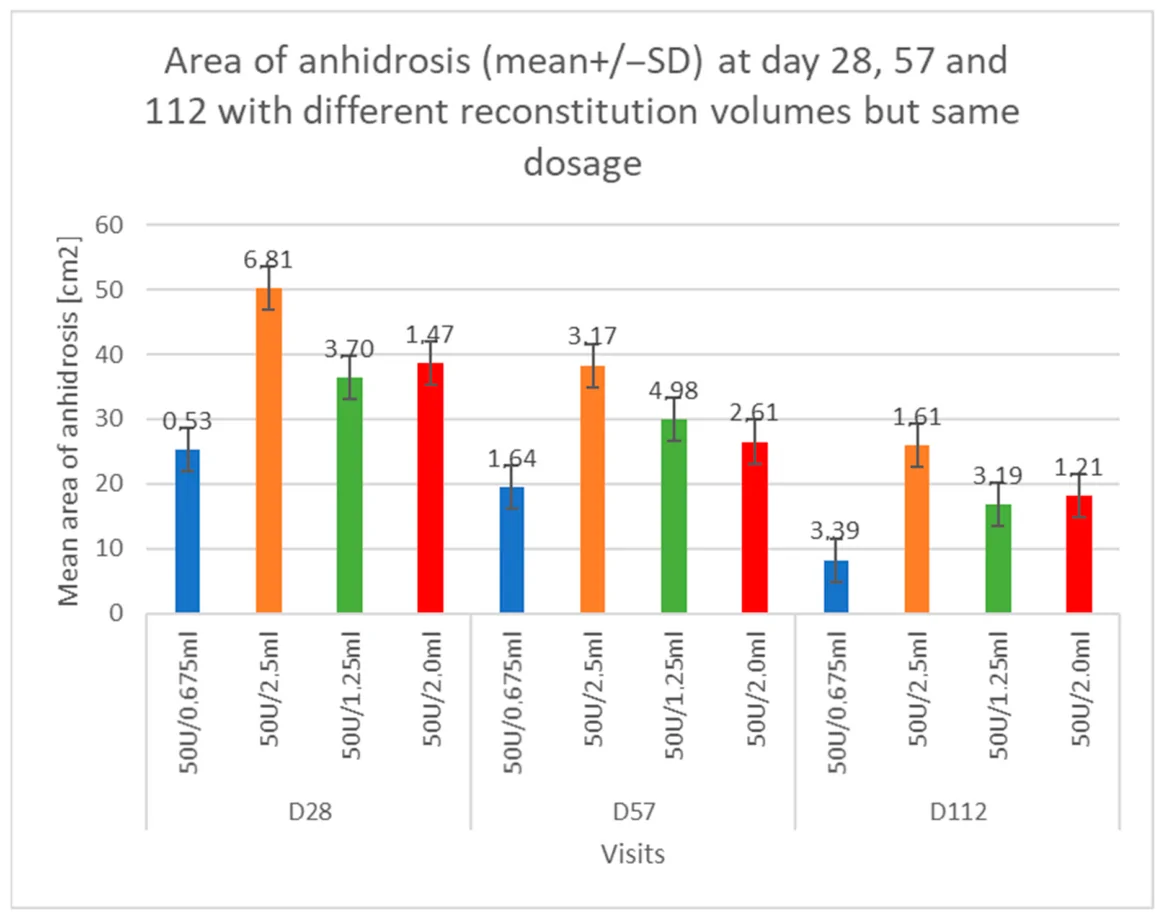
Dilution/Injection Volume Variation: Area of anhidrosis (mean ± SD, N = 3 subjects per group) at day 28, 57 and 112 with reconstitution volumes of 0.675–2.5 mL per 50 U vial at a constant unit dose of 4 U per injection point (4 injection points).

**Figure 3:**
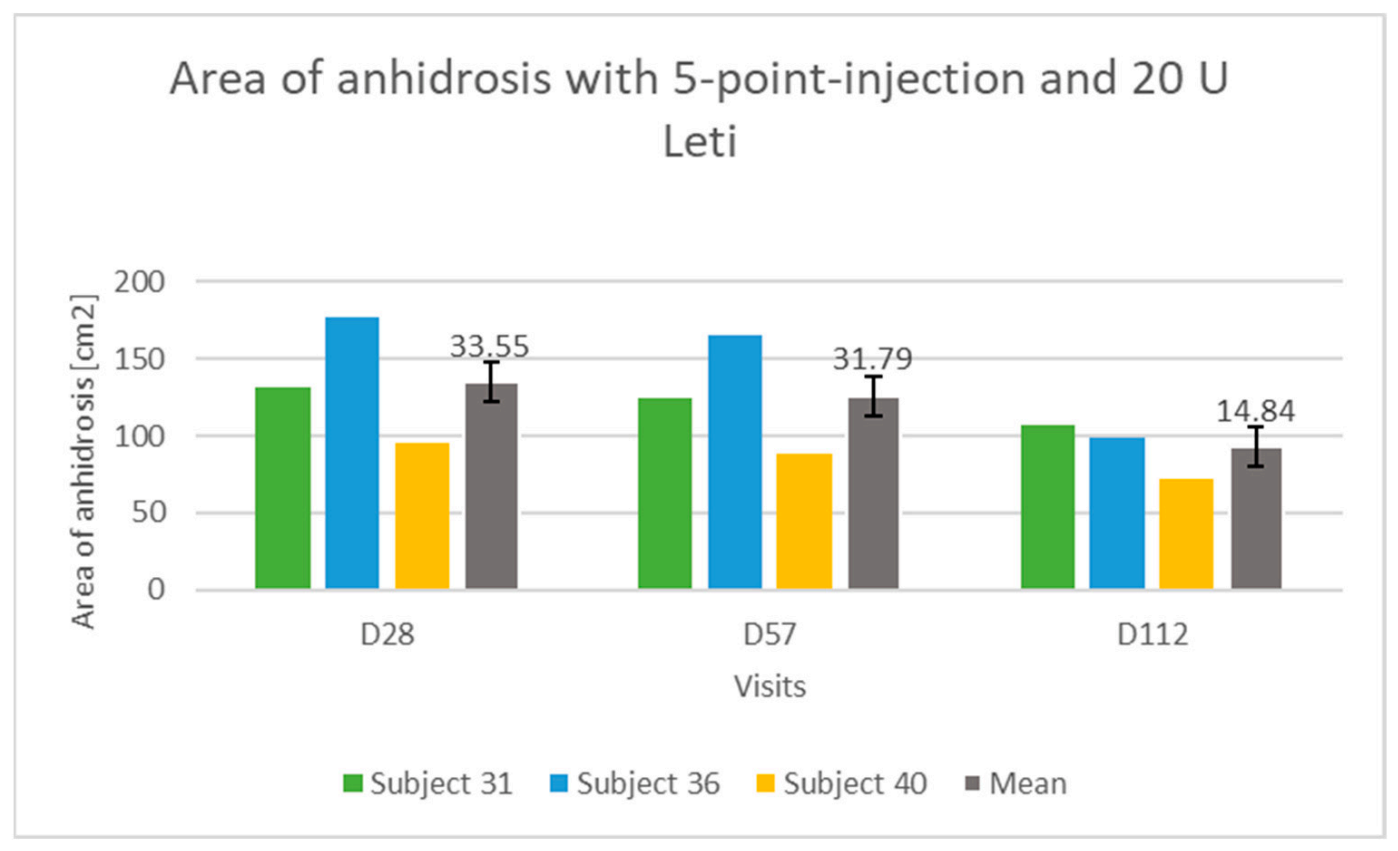
Mean diffusion area (±SD, N = 3 subjects) with the conventional 5-point injection pattern and a total dose of 20 U Leti-BoNTA, at day 28, 57 and 112.

**Figure 4:**
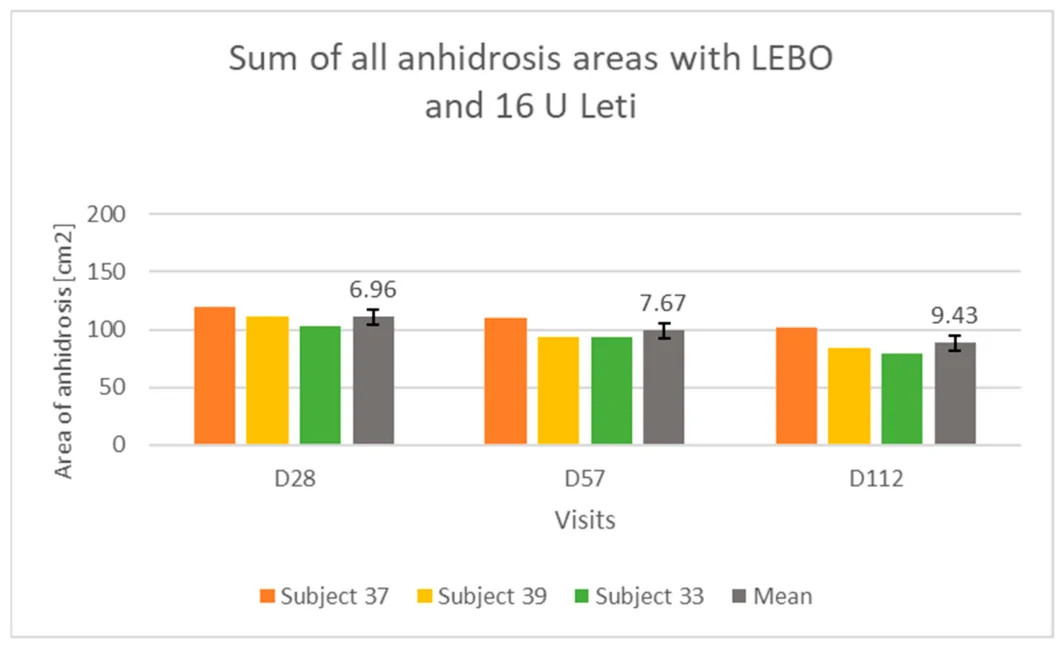
Sum of all anhidrosis areas (±SD, N = 3 subjects) with the LEBO injection pattern [7] and a total dose of 16 U Leti-BoNTA, at day 28, 57 and 112.

**Figure 5:**
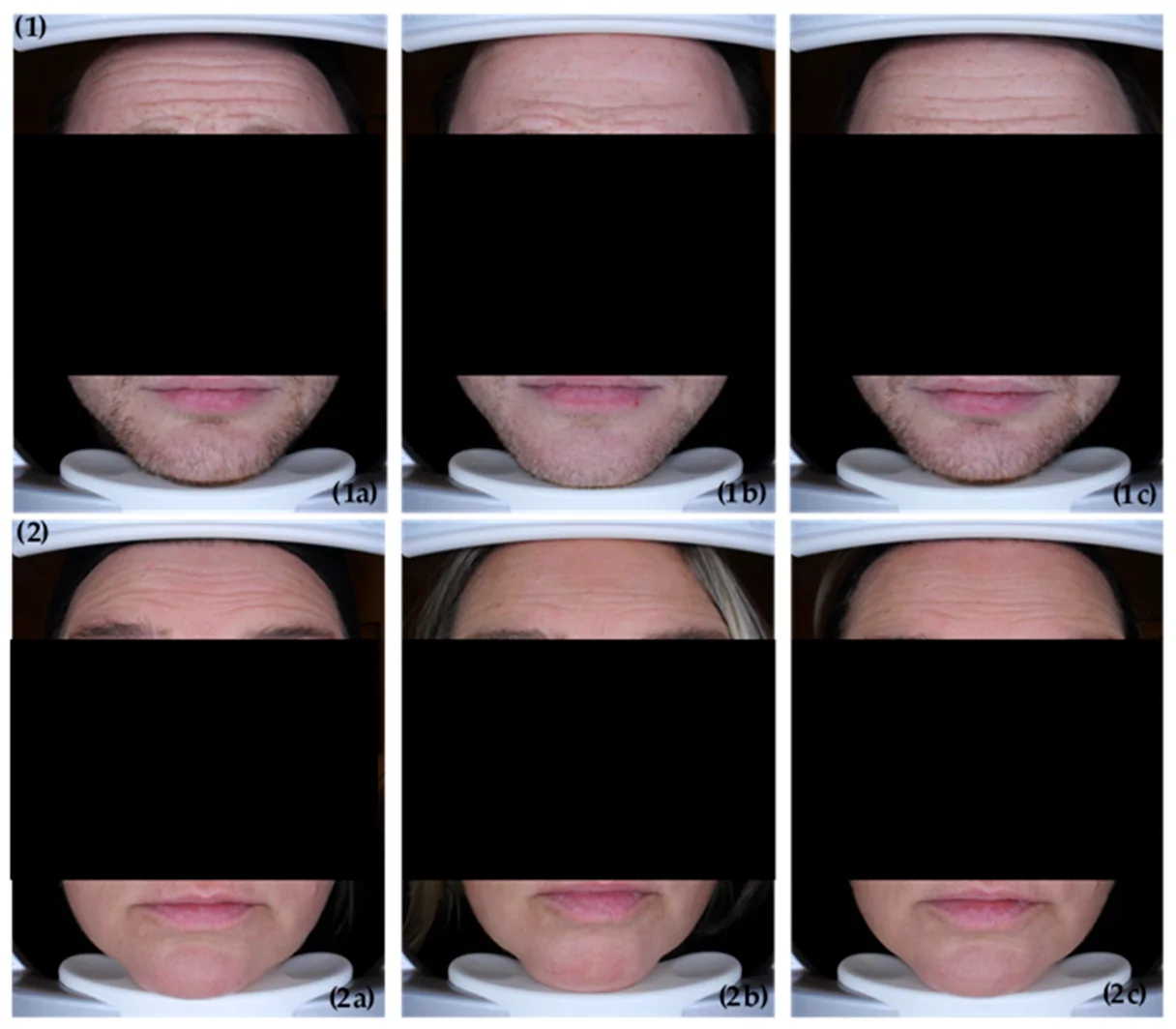
Standardized photographs of subjects during maximum frown following treatment with Leti-BoNTA at day 28 (**1a**,**2a**), 57 (**1b**,**2b**), and 112 (**1c**,**2c**). Recognizable facial expressions and individual facial characteristics were maintained throughout the study—(**1**) subject was injected with the 5-point injection pattern and (**2**) with the LEBO [7] injection pattern.

**Figure 6:**
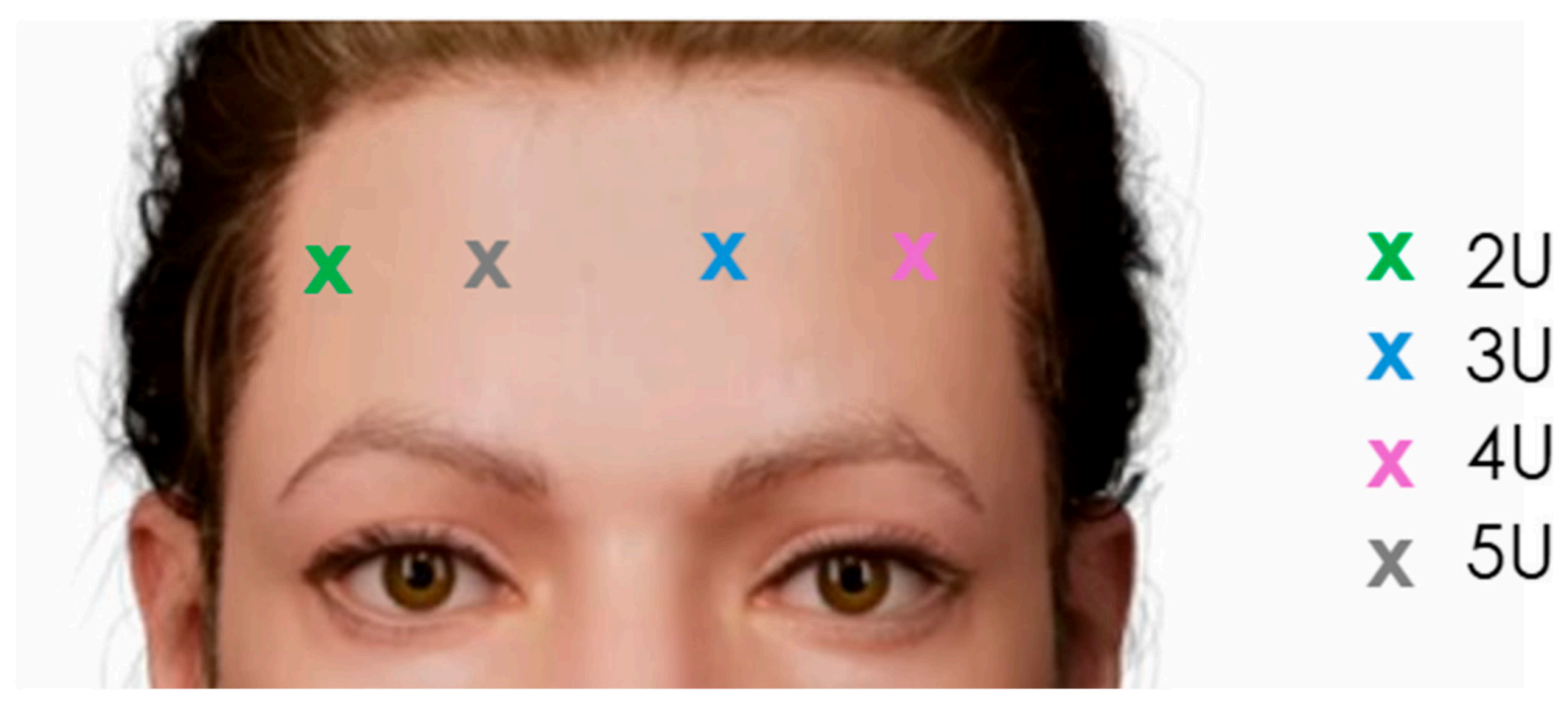
Injection template for Case 1.

**Figure 7:**
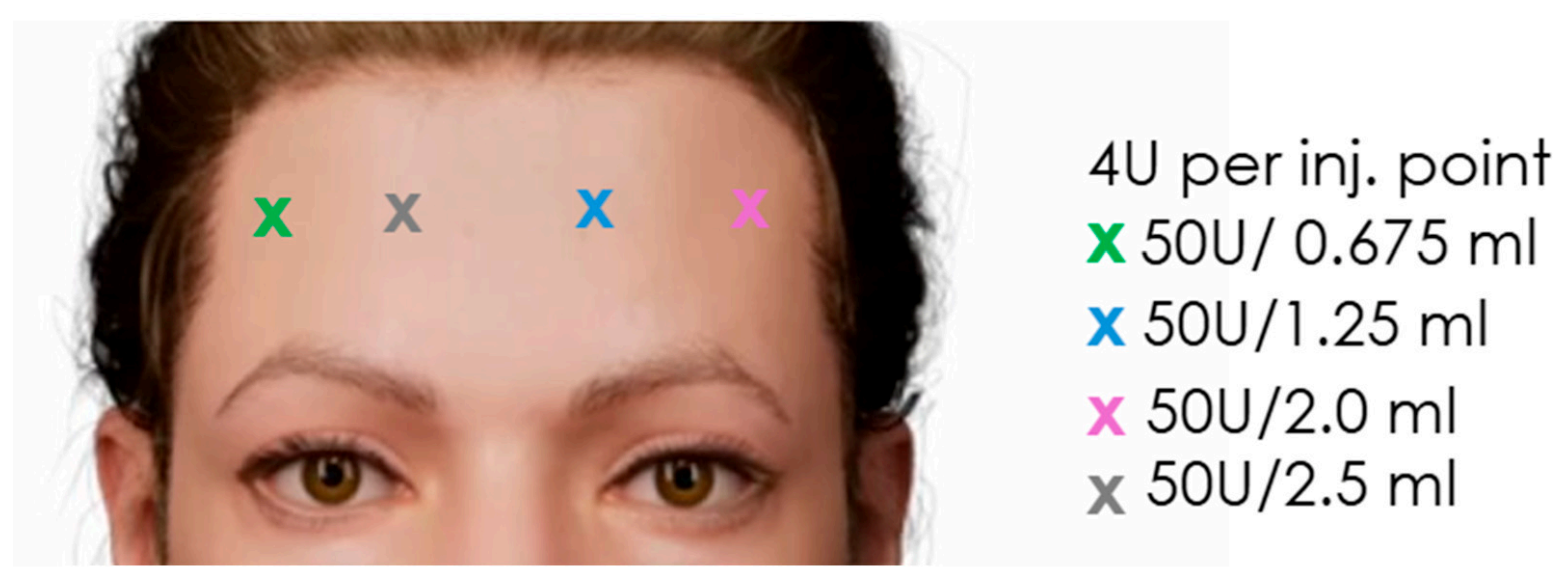
Injection template for Case 2.

**Figure 8:**
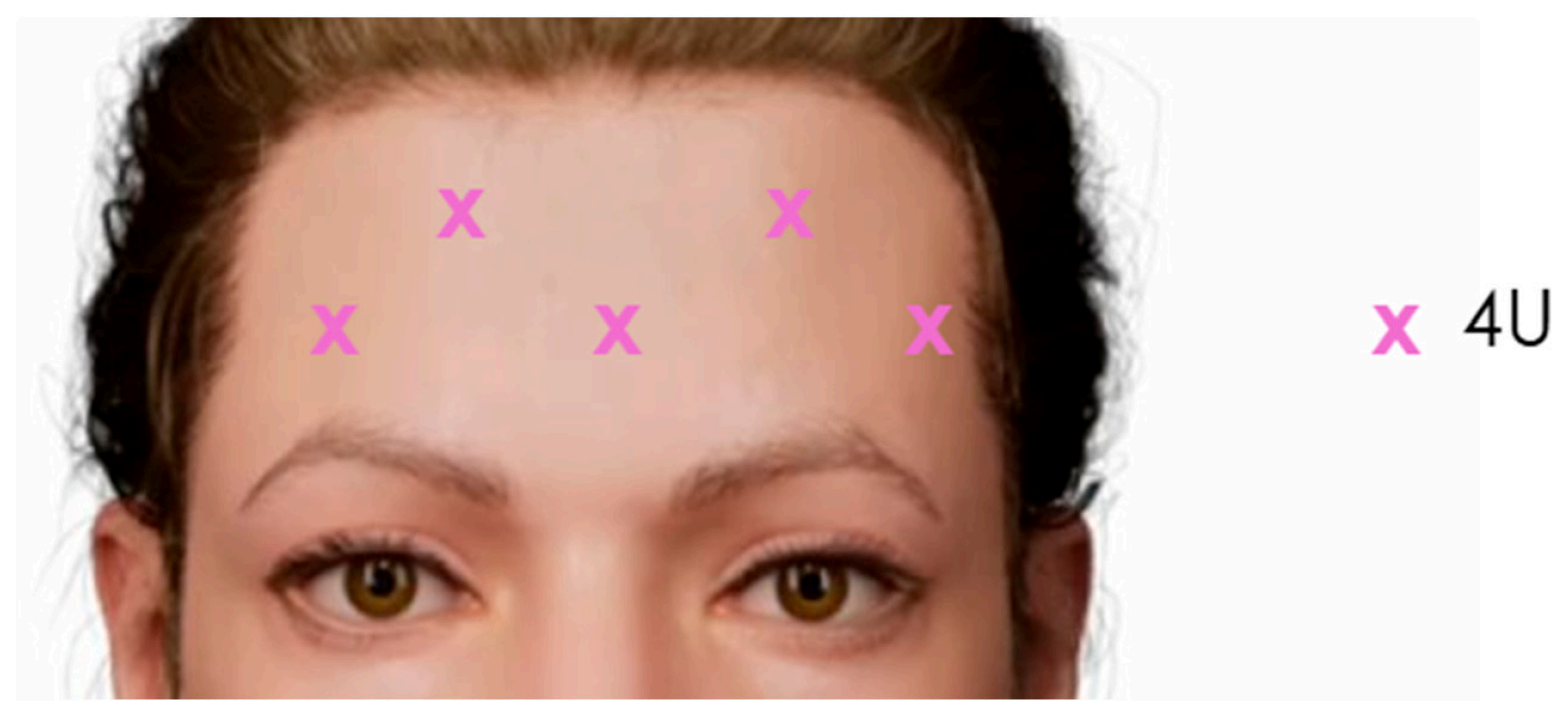
Injection template for Case 3.

**Figure 9:**
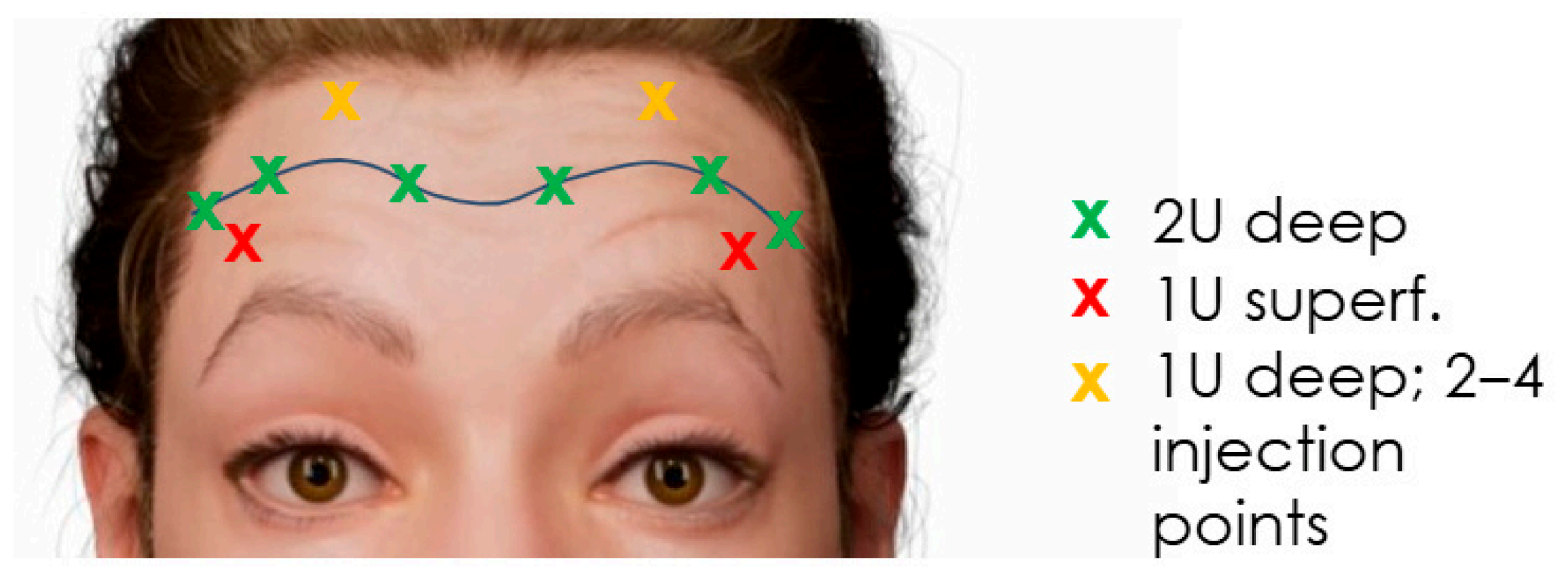
Injection template for Case 4.

**Table 1:** Anhidrotic area (mean ± SD, cm^2^) by injected dose at days 28, 57, and 112 (Case 1, dose variation).

Dose	D28 (M ± SD)	D57 (M ± SD)	D112 (M ± SD)
**2 U**	**14.49 ± 3.24**	**10.88 ± 3.87**	**6.58 ± 3.02**
**3 U**	**19.68 ± 7.09**	**15.63 ± 4.15**	**9.63 ± 2.78**
**4 U**	**27.22 ± 2.94**	**20.95 ± 2.51**	**13.48 ± 3.23**
**5 U**	**32.61 ± 2.88**	**27.70 ± 3.68**	**19.76 ± 1.44**

**Table 2:** Anhidrotic area (mean ± SD, cm^2^) by reconstitution volume at days 28, 57, and 112 (Case 2, dilution variation).

Reconstitution	D28 (M ± SD)	D57 (M ± SD)	D112 (M ± SD)
**0** **.** **675 mL**	**25.35 ± 0.53**	**19.53 ± 1.64**	**8.24 ± 3.39**
**1** **.** **25 mL**	**36.40 ± 3.70**	**29.92 ± 4.98**	**16.84 ± 3.19**
**2** **.** **0 mL**	**38.64 ± 1.47**	**26.53 ± 2.61**	**18.19 ± 1.21**
**2** **.** **5 mL**	**50.19 ± 6.81**	**38.33 ± 3.17**	**25.997 ± 1.61**

**Table 3:** Mean anhidrotic area ± SD (cm^2^) at days 28, 57, and 112 for the five-point injection pattern (Case 3) and the LEBO injection pattern (Case 4).

Case	D28 (M ± SD)	D57 (M ± SD)	D112 (M ± SD)
**5-point injection (Case 3)**	**134.94 ± 33.55**	**126.29 ± 31.79**	**93.14 ± 14.84**
**LEBO (Case 4)**	**111.17 ± 6.96**	**98.90 ± 7.67**	**88.48 ± 9.43**

**Table 4:** Injection paradigms used to investigate the influence of injection pattern, dose, and dilution of Leti-BoNTA.

Case	Injection Pattern	Injection Points	Reconstitution Volume with a 50 U Vial (mL)	Injection Volume per Point (mL)	Units per Injection Point (U)	Units in Total (U)
1	Dose variation	4	1.25	0.05/0.075/0.10/0.125	2/3/4/5	14
2	Dilution variation	4	0.675/1.25/2.0/2.5	0.054/0.10/0.16/0.20	4	16
3	5-point injection pattern	5	1.25	0.10	4	20
4	LEBO injection pattern [7]	10	1.25	Lateralization/Elevation points: 0.05 Balancing points: 0.025 Optimization points: 0.025	6 lateralization/elevation points: 2 U each, deep 2 balancing points: 1 U each, superficial 2 optimization points: 1 U each, deep	16

## Data Availability

The datasets generated and analyzed during the current study are not publicly available due to participant privacy and confidentiality agreements but may be available from the corresponding author on reasonable request.

## Abbreviations

The following abbreviations are used in this manuscript:

BMI	Body Mass Index
BoNTA	Botulinum Neurotoxin Type A
C-line	Line of Convergence
CT	Clinical Trial
EU	European Union
GCP	Good Clinical Practice
Leti-BoNTA	LetibotulinumtoxinA
U	Units
